# A New Image Enhancement and Super Resolution technique for license plate recognition

**DOI:** 10.1016/j.heliyon.2021.e08341

**Published:** 2021-11-10

**Authors:** Abdelsalam Hamdi, Yee Kit Chan, Voon Chet Koo

**Affiliations:** Multimedia University, Bukit Beruang Ayer Keroh, Melacca, 75450, Melacca, Malaysia

**Keywords:** Image Super Resolution, Image Enhancement, LPR

## Abstract

License Plate Recognition (LPR) is an important implemented application of Artificial Intelligence (AI) and deep learning in the past decades. However, due to the low image quality caused by the fast movement of vehicles and low-quality analogue cameras, many plate numbers cannot be recognised accurately by LPR models. To solve this issue, we propose a new deep learning architecture called D_GAN_ESR (Double Generative Adversarial Networks for Image Enhancement and Super Resolution) used for effective image denoising and super-resolution for license plate images. In this paper, we show the limitation of the existing networks for image enhancement and image super-resolution. Furthermore, a feature-based evaluation metric called Peak Signal to Noise Ratio Features (PSNR-F) is used to evaluate and compare performance between different methods. It is shown that the use of PSNR-F has a better performance indicator than the classical PSNR-pixel-to-pixel (PSNR-pixel) evaluation metric. The results show that using D_GAN_ESR to enhance the license plate images increases the LPR accuracy from 30% to 78% when blur images are used and increases the accuracy from 59% to 74.5% when low-quality images are used.

## Highlights

• Double Generative Adversarial Networks for Image Enhancement and Super Resolution.
• Improving the accuracy of LPR systems using image super resolution.

## Graphical abstract

**Figure:**
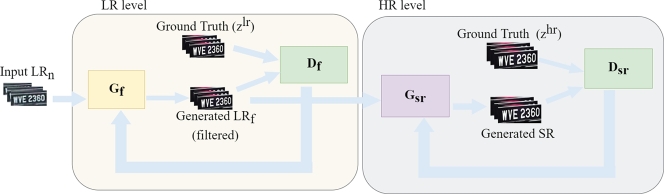

## 1 Introduction

**Figure 1:**
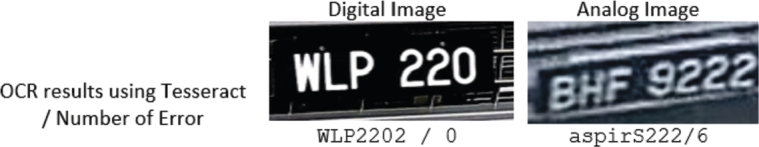
OCR comparison between High Resolution Digital camera and Low resolution Analog camera when Tesseract is used. Tesseract is an open source Long Short Term Memory (LSTM) [6] network for OCR, developed and trained by Google [7].

Computer vision applications have been widely used in the past few years. The capabilities of Artificial Neural Network (ANN) and Convolutional Neural Network (CNN) [1] shown in the past decades made it possible to translate pixels values to actions designed by developers. One of the commonly used computer vision applications is the License Plate Recognition (LPR) [2], [3], [4]. LPR is a computer vision application that automatically recognises the license plate characters from an image and converts them to editable text. LPR has a lot of essential applications nowadays. For example, searching an entire street looking for one specific car in a few minutes would not be possible for humans. However, LPR systems could do that in seconds [5]. LPR is also used for intelligent parking systems and many other applications. LPR faces many challenges, as the Optical Character Recognition (OCR) accuracy is proportionally related to the quality of the input image. For example, in Fig. 1, it is shown that the OCR engine fails to recognise the characters when the image resolution is low and noisy. The continuous movement of the car and the speed make it quite challenging to capture a clear image of the License Plate (LP). Not to forget the use of low budget analogue cameras in many security systems, which have a meagre resolution and bad quality in comparison to high priced digital cameras Fig. 1. Other limitations that affect the results are environmental factors such as light, dust, rain, etc. Many other measures are taken to overcome these limitations, such as ensuring good lighting conditions, better camera quality, and close camera positioning to capture a clear image. However, these measures are not applicable for all applications and thus, cannot be implemented everywhere.

Most of the current LPR techniques rely on a single image captured to retrieve the characters. However, these models do not work well for low quality and low-resolution images [8], [9], [10], [11].

**Figure 2:**
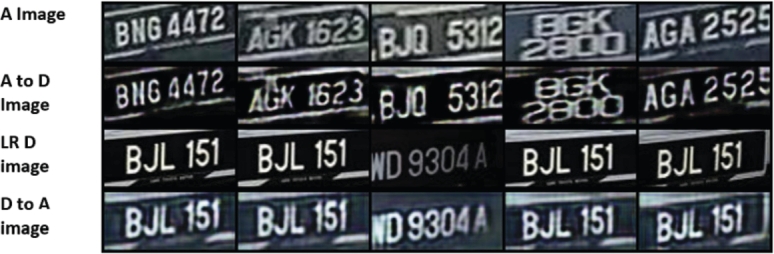
Generated Data using CycleGAN. Analog (A) Images are converted to Digital (D) Image and vice versa. The converted Analog with the original Digital images is used for the SISR training.

The past image enhancement research has shown that it is possible to improve image quality and image resolution using CNN and deep learning techniques [12]. Therefore, adding an image enhancer with a Single Image Super-Resolution (SISR) model using CNN will filter the image and enhance it, leading to more accurate and better results for LPR applications [13]. SISR is a task that maps a low-resolution (LR) image to a high-resolution (HR) image. Image enhancement techniques have been a hot research area for the past few years, especially after the first paper on the topic was published SRCNN [12], which utilised a CNN network to improve image resolution. The accuracy of SRCNN was very impressive when it was first published. Since then, a lot of research was published in the area, producing much better results. This is an excellent opportunity to implement such models to enhance the LP images. However, existing algorithms cannot enhance the image quality and increase the resolution simultaneously when the input image is boisterous, as shown in the fourth section of this paper. This paper proposes a Double Generative Adversarial Network (GAN) for image Enhancement and Super Resolution (D_GAN_ESR) to improve the LR images using a deep learning system. Our deep learning system is based on GAN architecture [14]. D_GAN_ESR consists of two concatenated GAN networks. The first GAN network is used for denoising and deblurring while maintaining the output image's resolution the same as the input image. The second GAN network is then used for Super-Resolution (SR), where the output image is four times larger than the input image. Recent studies have shown exemplary performance in mapping LR images to HR images using SISR. However, the studies did not consider mapping a distorted LR image to an HR image. A few studies applied some blurring filters with additional noise. However, the distortion added is not as complex as the noise due to analogue cameras. This paper proposes a more realistic approach in generating the LR images from HR images for training. First, the LR images are obtained by downsampling the HR images using the bicubic interpolation method. LR images are then distorted using an image-to-image style translation network such as CycleGAN [15] network, which will add more complex noise that looks exactly like the noise from the analogue camera Fig. 2.

The second section of this manuscript shows the previous related work of SISR, LPR Image SR, and image-to-image translation. The third section discusses the proposed methodology, including network architecture, the loss functions, and the hyperparameters of the deep learning model. The SISR results and LPR results before and after using our proposed method are shown in Section Four. The final section concludes the research done in this manuscript.

## 2 Related work

### 2.1 Single Image Super Resolution

SISR has been studied and applied in a variety of projects for the past decades. Earlier approaches rely on pure image processing such as bilinear interpolation, nearest-neighbour interpolation, bicubic interpolation, and other interpolation-based [16]. Some other approaches like natural image statistic [17], [18], Pre-defined Models [19]. A deep convolutional network has recently shown explosive popularity and powerful capability in mapping LR image to HR image [20], [21]. The first study was named SRCNN performed by Dong [12] which trains a CNN network consisting of three convolutional layers and aims to reduce the standard Mean Square Error (MSE) [22] loss between the output image and the ground truth. The end-to-end mapping between LR and HR using SRCNN [12] has achieved the state-of-the-art. Since then, many architectures have been proposed for SISR. A Very Deep Network (VDSR) [23] that learns a residual image was proposed using 16 convolutional layers inspired by the VGG [24] network for image classification. VDSR outperformed the SRCNN and proved that using a deep network was possible after SRCNN mentioned the disability for the network to learn when more than three convolutional layers are added. Nonetheless, VDSR style architecture requires upscaled images using any interpolation method as the input, which leads to heavier computation time and memory compared to the architectures with scale specific upsampling methods [21], [25], [26]. A deeper SR model termed SR-DensNet [27] was proposed using Densely Connected Convolutional Networks (DensNet) [28]. Unlike the previous methods, the input to the SR-DensNet was the LR image without any up-sampling method applied, which lowers the computational power. The feature maps generated from each layer were propagated to the next layer and every subsequent layer. All the generated feature maps are used at the last reconstruction layer to generate an HR image. This allows the construction layer to generate images using all the high and low-frequency feature maps. Although SR-DenseNet does not use an upsampled image as an input, it is still high computational expensive. To speed up the performance of SR tasks, FSRCNN [25] was proposed. By extracting feature maps from the low-resolution space and reconstruct an HR image at the last layer using transposed-convolution and sub-pixel convolution layers, respectively. FSRCNN [25] aims to reduce the MSE loss between the output image and the ground truth, like the previous methods. It was observed that reducing the MSE loss between the reconstructed HR image and the ground truth resulted in an overly smooth image. SRGAN [21] was proposed to overcome this issue by proposing for the first time an Adversarial Loss to the SR tasks.

The GAN [14] networks are widely used for multiple applications and have provided a way for unsupervised learning where labels do not exist by introducing an adversarial loss function. The architecture of GAN consists of two networks, the first network is where the image is generated, and it is called the generator. The second network will try to distinguish between the generated image and the real image, and it is called the discriminator. The generator will try to fool the discriminator by generating almost indistinguishable images from the actual data. SRGAN has shown that including the adversarial loss will result in a better and more realistic HR image quality. The SRGAN has also included the perceptual loss [29], [30] which aims to reduce the MSE between the feature maps of the reconstructed HR image and the ground truth generated by the VGG [24] network, instead of applying MSE to the final reconstructed image directly. Adding perceptual loss generates visually pleasing images which contain high-frequency details than the MSE-based methods. One of the best SISR methods is the EDSR [20] which is a modified version of SR-ResNet [21] inspired by ResNet architecture for Image classification [31].

### 2.2 LPR Image Super Resolution

Very little research has been focused on recovering an LP image using SR and image enhancement techniques. Although it is well known that LPR accuracy is proportionally related to the image quality. Many researchers are still using the earlier approaches such as Image processing and interpolation technique [32], [33]. Others focus on producing one HR image using multiple low-resolution images [34], [35]. A character semantic-based super-resolution for LR is proposed in [36]. However, it could be difficult to segment the characters if the image is corrupted with noise. In [37] a SISR for LP was proposed using an SRGAN and a perceptual OCR loss. Although perceptual OCR loss produced better character accuracy, the image quality was not more evident than SRGAN. Another limitation of this implementation is that the LR images used for training and testing were the downsampling using bicubic interpolation result from the HR image, with Gaussian noise and changing the colour of each pixel to black or white by chance of 10% (5% for each white and black). Although noise is introduced to the low-resolution image, it is different from the real noisy images where it is very random. In [38] another network based on SRGAN was also proposed. However, it only uses the bicubic LR image as an input without introducing any noise, which is not practical in a real-world application.

### 2.3 Image to image translation

Most of the current SISR methods rely on the bicubic downsampling images from the HR images. Some SISR methods introduce some Gaussian noise to blur the image, but in real-world scenarios, noise is not only obscured. The GAN [14] offers an excellent opportunity to produce non-existing data. Therefore, it can create a noisy fake date that looks precisely similar to the actual noisy data. GAN is extensively used to solve unsupervised learning when label images do not exist. Networks such as CycleGAN [15], and DualGAN [39] are excellent applications that perform image-to-image translation. Both networks consist of two generators. The first generator will map the image from domain X to domain Y. In contrast, the second generator will map the images back from domain Y to domain X to maintain a cycle consistency. The image-to-image translation networks are different from the SR application as their input, and the output is the same size, while in SR tasks, the results are several times larger than the input images. However, image-to-image translation offers an excellent opportunity to generate high distorted images that look exactly like the real-world distorted images by translating the output of the bicubic down sample of HR images to a real-world distorted image.

In this paper, a realistic image enhancement and SR method has been proposed using realistic data captured using a high-quality camera. Other low-quality data collected from the government were captured using low-quality analogue cameras. The HR images are downsampled and distorted using CycleGAN [15] network. Finally, the HR and the output LR data are used to train the D_GAN_ESR network.^1^

## 3 Methodology

Traditional SISR [40] formulates the convolutional task as $x=\mathrm{SH}z^{hr}+n$ where *x* and $z^{hr}$ are the noisy LR ($LR_{n}$) image and the Ground truth HR image, respectively. S and H are the kernel size and blurring matrix, respectively. Where *n* is randomly added noise. A more general formulation was proposed in [41] $x=f_{n}(f_{d}(z^{hr}))+n$, where $f_{d}$ is the downsampling function and $f_{n}$ is a degradation function where blurring and shifting noises are added. However, other noises might be included in real-world scenarios, such as colour noises, especially when using analogue cameras where the resolution is low, and the colour is distorted. In this paper, a new formulation which is generally the same as the one in [41] is proposed, except that our $f_{n}$ is a convolutional neural network to generate high distorted images that look like analogue images. Thanks to image-to-image translation using CycleGAN [15], high distorted images have been generated. The final formulation of our LR image is $x=f_{cycleGAN}(f_{d}(z^{hr}))$. No additional noises are added as the image is noisy enough Fig. 2.

**Figure 3:**
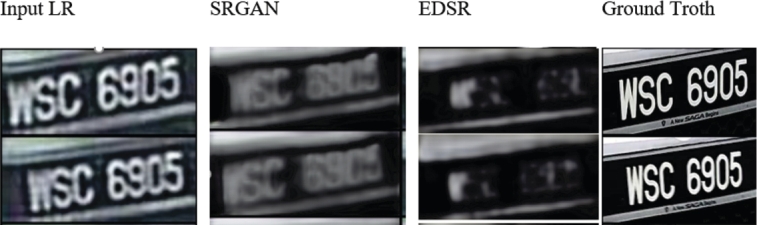
Experimenting on different image super-resolution methods.

The difference between SISR and image-to-image translation is that SISR accepts LR images and output an HR image of several times larger resolution while the image-to-image translation outputs an image of the same size as the input. SISR requires the output image to be of higher quality, unlike the image-to-image translation where only a different style is obtained [41]. Using image-to-image translation for the SR task will be difficult as upsampling the image using interpolation methods is needed, leading to amplifying the noise in the image. Applying the current SISR methods such as EDSR [20] and SRGAN [21] directly as in Equ. (1) to the LR image has failed to obtain a clean HR image using a single forward function as shown in Fig. 3. It is difficult to enlarge the image resolution and clean a complex noise using a single CNN (Generator) network.

(1) $$y=G_{SR}(x)$$

where *y* is the output HR image, *x* is the input $LR_{n}$ and $G_{SR}$ is the SR network.

Although both networks used the perceptual loss [29], [30] which is used to solve the regression to the mean problem [42] caused by the MSE loss function [43], the results are unpleasing. To overcome this limitation, a Double GAN for image Enhancement and Super Resolution (D_GAN_ESR) network is proposed. The first GAN network is used to learn a mapping from the $LR_{n}$ domain to the LR noise-free ($LR_{f}$) domain as represented in Equ. (2).

(2) $$y^{\prime }=G_{f}(x)$$

where *x* is the $LR_{n}$ image and $y^{\prime }$ is the $LR_{f}$ image with the same resolution as $LR_{n}$.

**Figure 4:**
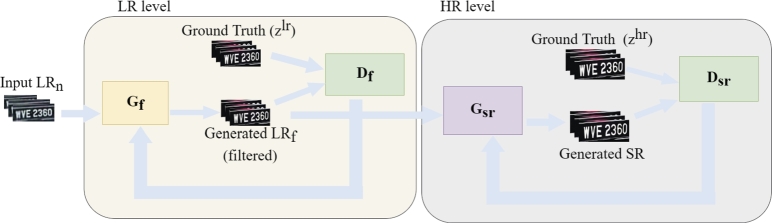
The architecture of the proposed method (D_GAN_ESR), where *G*_*f*_ is the generator to filter an image, and *G*_*sr*_ is a generator for SR. *D*_*f*_ and *D*_*sr*_ are the discriminator for adversarial loss, for *G*_*f*_ and *G*_*sr*_ respectively.

Another GAN network is then used to learn a mapping from $LR_{f}$ image to the HR image represented in Equ. (3).

(3) $$y=G_{SR}\left(y^{\prime }\right)$$

where y is output HR image and $y^{\prime }$ is the filtered image obtained in Equ. (2). Fig. 4 shows the overall D_GAN_ESR architecture for mapping LR noisy image to HR image. The end-to-end mapping from *x* to *y* is represented in Equ. (4)

(4) $$y=G_{SR}\left(G_{f}(x)\right)$$

### 3.1 Image filtering

The first part of the D_GAN_ESR architecture is responsible for cleaning all the noise of the LR image. The input to the network is noisy $LR_{n}$, and the output of the network is the clean $LR_{f}$.

**Figure 5:**
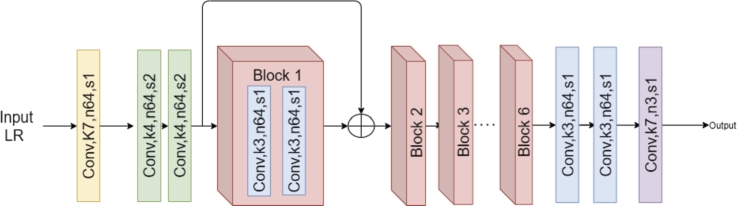
The architecture of *G*_*f*_ where Conv represent a convolutional layer, *k* is the kernel size, *n* is the output feature maps, and *s* is the stride.

**Generator network:** The generator network consists of three convolutional layers for feature extraction, followed with six residual blocks [31], each block consists of two convolutional layers. Finally, a reconstruction block consists of three convolutional layers is added. The generator network is shown in Fig. 5.

**Figure 6:**
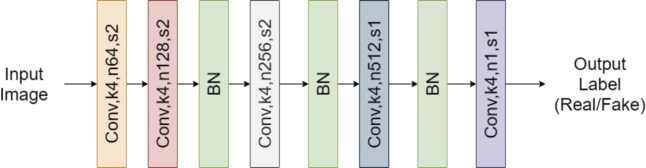
The architecture of *D*_*f*_ where Conv represent a convolutional layer, *k* is the kernel size, *n* is the output feature maps, and *s* is the stride. BN represents a batch normalisation layer.

**Discriminator network:** The discriminator network consists of five convolutional layers, with a Batch Normalisation (BN) layer between each convolutional layer. The discriminator network is shown in Fig. 6.

**Loss functions:** The adversarial loss is used to ensure smoothness and realistic image prediction. To make the training more stable, the least square error is used for the adversarial loss Equ. (5) [41].

(5) $$L_{A}^{lr}=\frac{1}{N}\sum_{i}^{N}{\left\|D_{f}^{lr}\left(G_{f}^{lr}\left(x_{i}\right)\right)-1\right\|}_{2}$$

where *N* is the number of training samples, $G_{f}^{lr}$ and $D_{f}^{lr}$ are the generator and the discriminator for the filtering network at the LR level, respectively. $L_{A}^{lr}$ is the adversarial loss at the LR level. The VGG-19 [24] network is used for perceptual loss [29], [30] implementation, where the extracted features of the output image and the clean low-resolution image are obtained and compared using the MSE function. The perceptual loss function is represented in Equ. (6)

(6) $$L_{P}^{lr}=\frac{1}{N}\sum_{i}^{N}{\left\|vgg_{19}\left(G_{f}^{lr}\left(x_{i}\right)\right)-vgg_{19}\left(z_{i}^{lr}\right)\right\|}_{2}$$

where $L_{P}^{lr}$ is the perceptual loss, $vgg_{19}$ is the network to extract the feature maps, and $z^{lr}$ is the downsampled image from the ground truth $z^{hr}$ using bicubic interpolation without any noises added.

In addition, the identity loss for image-to-image translation proposed in [15] and represented in Equ. (7) is used. It is shown that identity loss helps to preserve colour composition between input and output images.

(7) $$L_{I}^{lr}=\frac{1}{N}\sum_{i}^{N}{\left\|\left(G_{f}^{lr}\left(z_{i}^{lr}\right)\right)-z_{i}^{lr}\right\|}_{1}$$

where $L_{I}^{lr}$ is the identity loss at the LR level. The Least Absolute Deviations loss function known as L1 [22], [44] has been added to improve the PSNR-Pixel. Our several tries showed that using the L1 loss function represented in Equ. (8) makes the training more stable and easier for the network to converge than the MSE loss function.

(8) $$L_{L1}^{lr}=\frac{1}{N}\sum_{i}^{N}\left({\left\|\left(G_{f}^{lr}\left(x_{i}\right)\right)-z_{i}^{lr}\right\|}_{1}\right)$$

where $L_{L1}^{lr}$ is the L1 loss at the LR level. The overall loss function mapping $LR_{n}$ to $LR_{f}$ is presented in Equ. (9).

(9) $$L_{f}^{lr}=\alpha_{1}L_{A}^{lr}+\alpha_{2}L_{P}^{lr}+\alpha_{3}L_{I}^{lr}+\alpha_{4}L_{L1}^{lr}$$

where $\alpha_{1}$, $\alpha_{2}$, $\alpha_{3}$ and $\alpha_{4}$ are the weight factors for each loss at the LR level.

### 3.2 Image Super Resolution

For the SISR task, the same generator as the EDSR [20] network SISR is used, followed by a discriminator network similar to $D_{f}^{lr}$. The loss functions for the SR network are the same as the loss functions used in the LR level, just that $G_{f}^{lr}$ and $D_{f}^{lr}$ are replaced to be $G_{sr}^{hr}$ and $D_{sr}^{hr}$ respectively. Where $G_{sr}^{hr}$ and $D_{sr}^{hr}$ are the generator and the discriminator for the SR task at the high-resolution level, only the identity loss is changed as the input and output must be of the same size. To solve this, the identity loss proposed in [41] is used and represented in Equ. (10).

(10) $$L_{I}^{sr}=\frac{1}{N}\sum_{i}^{N}{\left\|\left(G_{sr}^{hr}\left(z_{i}^{lr}\right)\right)-z_{i}^{hr}\right\|}_{2}$$

where the $z^{lr}$ is the LR image obtained by downsampling the ground truth HR image and $z^{hr}$ image is the ground truth. The total loss mapping $LR_{f}$ to HR is given in Equ. (11).

(11) $$L_{f}^{sr}=\omega_{1}L_{A}^{sr}+\omega_{2}L_{P}^{sr}+\omega_{3}L_{I}^{sr}+\omega_{4}L_{L1}^{sr}$$

where $\omega_{1}$, $\omega_{2}$, $\omega_{3}$ and $\omega_{4}$ are the weight factors for each loss at the HR level.

### 3.3 Hyper parameters and training

A 1000 paired images are used for training and testing. Each pair consists of a coloured LR image and HR image. The resolution of the LR image is 92×40 while the HR resolution is four times larger, which is 368×160. To train the network Adam optimizer [45] is used with a learning rate of $2\times 10^{\left(-4\right)}$, and PyTorch default values of betas $\beta_{1}=0.5$, $\beta_{2}=0.999$, and $\mathrm{eps}=1\times 10^{\left(-8\right)}$. The learning rate is then minimised to $1⁎10^{\left(-4\right)}$ after 35 epochs of the training. Unlike the traditional SR methods, where the image is cropped into smaller parts, an LP image is not suitable to be cropped randomly to avoid cropping a part of any character. Therefore, the complete image is fitted to the generator with a size of 40×92 and outputs four times higher resolution image. The output image will be of a size 160×368. A batch size of 8 is selected and trained for 100 epochs. The values of $\alpha_{1}=1$, $\alpha_{2}=0.5$, $\alpha_{3}=5$ and $\alpha_{4}=3$ are selected for the LR filtering part. For the SR part $\omega_{1}=1$, $\omega_{2}=0.3$, $\omega_{3}=1$ and $\omega_{4}=5$ are selected.

## 4 Results

### 4.1 Enhancement and SR results

**Figure 7:**
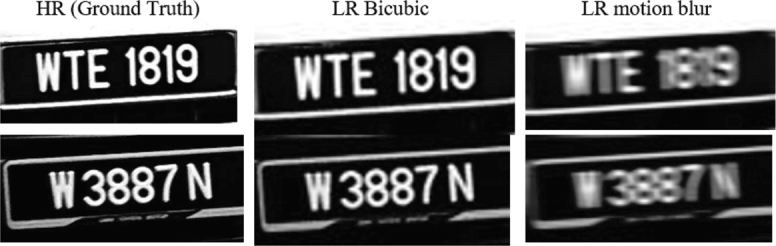
Sample from Dataset 1.

The proposed SISR results for LP images are compared with the following SISR methods, SRGAN [21], EDSR [20], and ESRGAN [46], using three evaluation metrics. The evaluation metrics are the classical Peak Signal To noise ratio pixel-to-pixel (PSNR-pixel) (usually it is referred to as PSNR only, but we add the word pixel to avoid confusion with other PSNR comparison methods), Structure Similarity Matrix (SSIM), and PSNR Feature (PSNR-F). PSNR-F is the PSNR between the feature maps of the HR image and SR image extracted using the VGG-19 network. The higher the PSNR-F, the more features are recovered. It is shown that PSNR-F has a better performance indicator as compared to PSNR-pixel. We retrain SRGAN, EDSR, and ESRGA models for a fair benchmark, including D_GAN_ESR (our network), using two datasets. The first one is where a motion blur noise with kernel size five is added to the resized bicubic image with a factor of 4. Fig. 7 shows an example of dataset 1.

**Figure 8:**
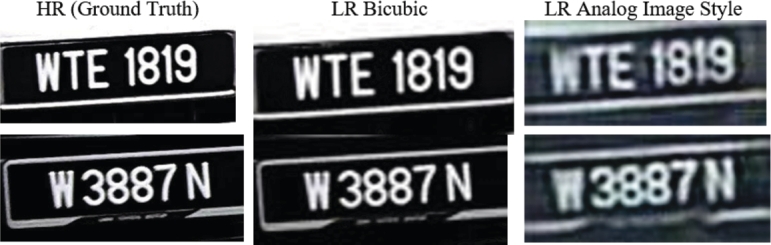
Sample of Dataset 2.

The second dataset where a CycleGAN [15] is used to transfer the style to an analogue image style, which introduced blur, shifting and colour noises to the image. Fig. 8 shows an example of dataset 2.

The results show that the new proposed network for LP dataset 1 obtains a comparable result with the current SISR algorithms. Table 1 shows the comparison in terms of PSNR-Pixel, SSIM, and PSNR-F.

**Table 1:** Quantitative evaluation on dataset 1 of the proposed architecture in terms of PSNR-Pixel, SSIM, and PSNR-F in comparison with SRGAN, EDSR and ESRGAN.

	Bicubic	SRGAN	EDSR	ESRGAN	D_GAN_ESR (Ours)
PSNR-pixel	30.182	31.570	**32.328**	29.904	31.286
SSIM	0.508	0.784	**0.829**	0.667	0.781
PSNR-F	−7.013	0.791	−0.876	**1.023**	−0.423

**Figure 9:**
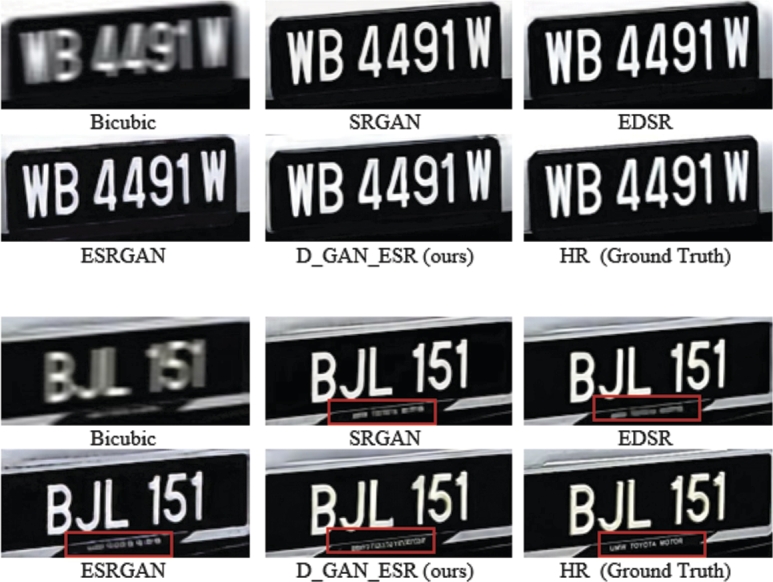
Visual Comparison between SRGAN, EDSR, ESRGAN and D_GAN_ESR results when dataset 1 is used.

**Figure 10:**
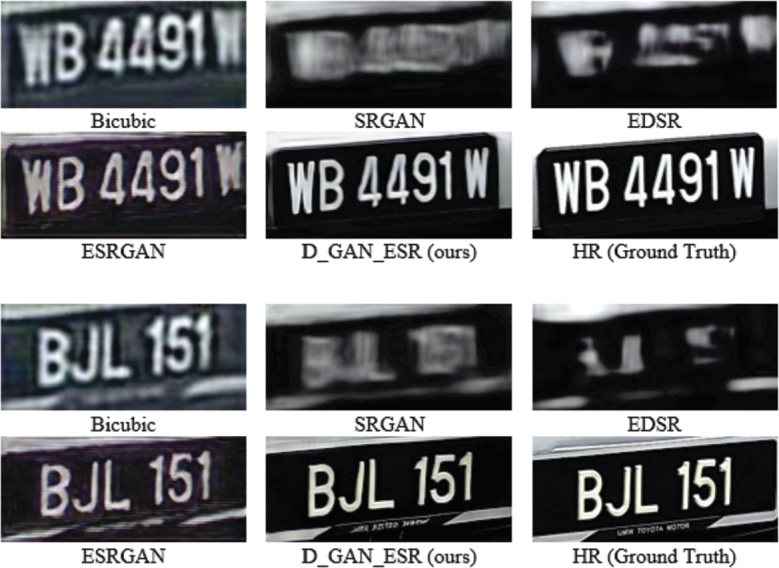
Visual Comparison between SRGAN, EDSR, ESRGAN and D_GAN_ESR results when dataset 2 is used.

Although our PSNR-Pixel, SSIM, and PSNR-F results are not the highest, the results of all the networks provide a great visually pleasing image Fig. 9. Notice that our methodology can recover some features (Highlighted by the red box in Fig. 9) that are not recovered by other networks (higher PSNR and SSIM does not always mean better feature recovering or visually better images [21]). These features are not very clear, but they indicate that our network focuses more on the features of the image than other networks. Although all networks could recover clear images when dataset 1 is used, this is not the case when dataset 2 is used. Table 2 shows the PSNR-pixel, SSIM and the PSNR-F summary when dataset 2 is used, using the same networks. Few samples of results are shown in Fig. 10.

**Table 2:** Quantitative evaluation on dataset 2 of the proposed architecture in terms of PSNR-Pixel, SSIM, and PSNR-F and compared with SRGAN, EDSR and ESRGAN.

	Bicubic	SRGAN	EDSR	ESRGAN	D_GAN_ESR (Ours)
PSNR-pixel	27.745	28.331	29.095	27.703	**29.558**
SSIM	0.094	0.204	**0.271**	0.067	0.227
PSNR-F	−5.913	−8.753	−8.530	−3.171	−**2.653**

The results show that our proposed network provides the maximum PSNR-Pixel and visually realistic image recovery. Although EDSR resulted in a high PSNR-Pixel, the image is far from the HR image. Therefore, adding a different comparison method is essential. D_GAN_ESR network has achieved the highest PSNR-F. Compared to ESRGAN, the PSNR-F is close, but our results are much higher in PSNR-Pixel. D_GAN_ESR network could perform better due to the usage of two GAN networks by filtering the image in the low-resolution level and then applying the SR. Using SR directly to an image will make it difficult to train one generator to filter a massive noise and increase the resolution of an image to several times higher in a single forward network.

### 4.2 LPR results

As mentioned previously, the accuracy of the LPR systems is proportionally related to the quality of the input image. In this section, a comparison between the LPR results before and after the use of D_GAN_ESR. Two OCR engines are used for comparison, which are Tesseract [7] and easyOCR [47] [48] [49]. Both engines utilise Long Short Term Memory (LSTM) [6] deep learning network for OCR detection.

The LPR testing results before and after using the D_GAN_ESR network for dataset 1 and dataset 2 are shown in Table 3. The average error rate when dataset 1 is used has improved after using D_GAN_ESR from 4.84 and 5.75 to 1.52 and 1.58 when Tesseract and easyOCR engines are used, respectively. However, for dataset 2, where low quality and low-resolution images are used, the OCR results have improved from 2.85 and 2.93 average error per image to 1.79 and 1.61 when Tesseract and easyOCR engines are used, respectively. The OCR error calculation is based on the edit distance algorithm [50]. The number of 100% correct predictions out of 195 testing images has increased after using D_GAN_ESR from 1 and 0 prediction to 69 and 53 predictions when dataset 1 is used with Tesseract and easyOCR engines, respectively. For dataset 2 the number of 100% correct predictions has increased from 16 and 11 to 54 and 60 when using Tesseract and easyOCR engines, respectively, as shown in Table 4.

**Table 3:** Average error rate per image of the LPR results before and after the use of the proposed architecture.

	Before Tessract	Before easyOCR	After Tesseract	After easyOCR
DataSet 1	4.84	5.75	1.52	1.58
DataSet 2	2.85	2.93	1.79	1.61

**Table 4:** Number of 100% correct predictions before and after using D_GAN_ESR.

	Before Tessract	Before easyOCR	After Tesseract	After easyOCR
DataSet 1	1	0	69	53
DataSet 2	16	11	54	60

A few examples of the LPR results before and after using D_GAN_ESR are shown in Table 5.

**Table 5:**
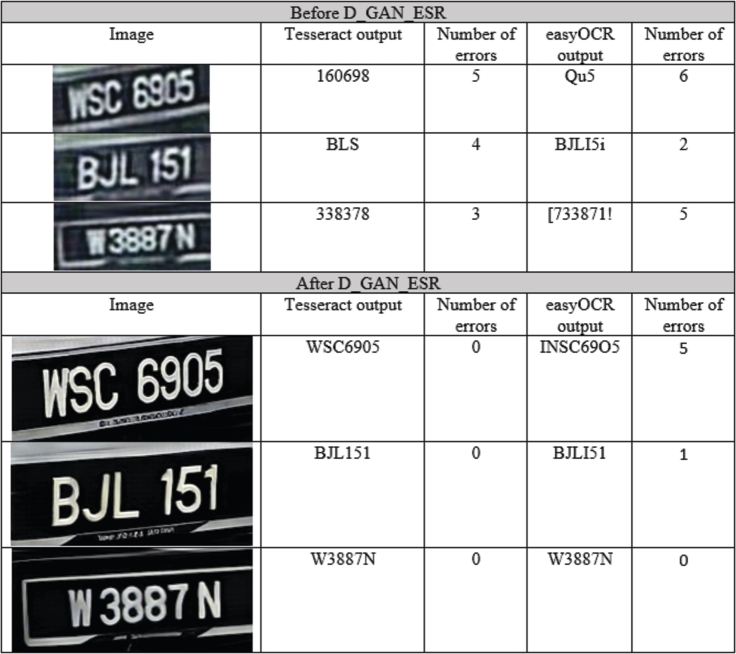
LPR results before and after the use of D_GAN_ESR.

### 4.3 Computation comparison

On the other hand, our network is more computationally expensive therefore, it takes a longer time during training. A comparison between the proposed method and existing methods in terms of the number of trainable parameters and processing time per iteration during training and testing are shown in Table 6. The total number of parameters in our model is less than ESRGAN. However, it has more parameters than SRGAN and EDSR. The iteration time during testing is proportionally related to the number of parameters. That is why D_GAN_ESR takes a longer time than SRGAN and EDSR during testing but takes less time than ESRGAN. During training, the number of loss functions and their complexity plays a role in the processing time. D_GAN_ESR takes a longer time during training than the rest of the algorithms due to the multiple loss functions used.

**Table 6:** Comparison between the proposed method and existing methods in terms of second per iteration during training and testing.

	SRGAN	EDSR	ESRGAN	D_GAN_ESR(ours)
Training time per iteration	0.5280s	0.0810s	0.4401s	**0.6382s**
Testing (Inference) time	0.0109s	0.0080s	**0.0520s**	0.0120s
Number of parameters	5948236	1517571	**10331843**	8935374

## 5 Conclusion

A new convolutional neural network architecture has been proposed to restore a highly distorted image to a better quality image with higher resolution. Our study shows that a single forward generator cannot remove complex noises and increase the resolution of an image concurrently. Although using two GAN networks increases the computational time during training, it provides a much cleaner image with a higher resolution. It is also shown that our network offers comparable performance under motion blur conditions. Our study showed that using the D_GAN_ESR network shows a notable enhancement on the results of the LPR models.

## Declarations

### Author contribution statement

Abdelsalam Hamdi: Conceived and designed the experiments; Performed the experiments; Analyzed and interpreted the data; Contributed reagents, materials, analysis tools or data; Wrote the paper.

Voon Chet Koo, Yee Kit Chan: Conceived and designed the experiments; Contributed reagents, materials, analysis tools or data.

### Funding statement

Abdelsalam Hamdi Abdelaziz was supported by Yayasan Universiti Multimedia.

### Data availability statement

Data will be made available on request.

### Declaration of interests statement

The authors declare no conflict of interest.

### Additional information

No additional information is available for this paper.

## Footnotes

1 The dataset used for this research is available upon request from the authors.
